# Deglution

**Published:** 1865-12

**Authors:** 


### DEGLUTION.

Some important physiological experiments on deglutition have just been made with the auto-laryngoscope by Dr. H. Guinier. In a note communicated to the French Academy, he states that in the process of swallowing, the solid food passes directly upon the surface formed by the contraction of the glottis. In the same manner, liquids employed as gargles were found to remain below the epiglottis, in direct contact with the folds of the intra-laryngeal mucous membrane and the vocal cords. From these experiments it appeared to follow that the simple contraction of the vocal cords is sufficient to oppose the passage of foreign bodies into the trachea. This contraction is automatic, and is produced by reflex action. This, in turn, is due to the sensation caused by the contact of the foreign substance with the membrane lining the regions under the glottis, but more especially under the epiglottis. This membrane, therefore, appears to play the part of a special sensory organ. — *Med. and Surg. Rep.*

